# Treatment of extensive oral leukoplakia with diode laser – Successful case report^؆^

**DOI:** 10.1016/j.abd.2025.501274

**Published:** 2026-01-15

**Authors:** Elen de Souza Tolentino, Fábio Vieira de Miranda, Tiago Carvalho dos Santos, Lucas Hideki Suzaki Ikeshoji, Letícia Sant’Anna Arioso, Paulo Sérgio da Silva Santos, Camila Lopes Cardoso

**Affiliations:** a Department of Dentistry, Universidade Estadual de Maringá, Maringá, PR, Brazil; b Faculty of Dentistry, UniCesumar, Maringá, PR, Brazil; c Department of Surgery, Stomatology, Pathology and Radiology, Bauru Faculty of Dentistry, Universidade de São Paulo, Bauru, SP, Brazil; d Graduation, Centro Universitário Sagrado Coração, Bauru, SP, Brazil; e Post-Graduation, Centro Universitário Sagrado Coração, Bauru, SP, Brazil

Dear Editor,

Oral leukoplakia (OL) is the most common potentially malignant disorder, defined as a non-removable white patch or plaque that cannot be clinically or microscopically characterized as another lesion; therefore, its diagnosis is made by exclusion.^1^ OL has a variable malignant transformation rate, between 3.5% and 9.5%.^2^, ^3^ After confirmation of its diagnosis through incisional biopsy, recommendations for discontinuing risk factors such as smoking and alcohol consumption, and the application of surgical or non-surgical therapeutic methods are given to achieve its resolution.^1^

Non-surgical approaches include the topical application of retinoids, bleomycin, cyclooxygenase inhibitors, and photodynamic therapy. However, these have been associated with a high recurrence rate, adverse effects, and a lack of scientific evidence.^1^ Surgical intervention should be performed, whenever possible, using conventional excision procedures with a cold scalpel or other technologies such as high-power laser, which has been extensively explored in the last decade.

In more extensive leukoplakia, the cold scalpel procedure becomes challenging due to the risk of bleeding and the infeasibility of joining the tissues by suturing. Given the aforementioned limitations, the diode laser emerges as a viable option, as it allows a precise incision, ensures hemostasis, and eliminates the need for suturing.^4^ Long-term studies on the prognosis of OL after surgical removal with a diode laser are scarce.^5^, ^6^, ^7^ This article describes a case of extensive OL, with high-grade epithelial dysplasia, in the buccal mucosa, successfully treated by surgical laser.

A 57-year-old male patient, a smoker (15 cigarettes a day for 43 years), showed, four years before, a painless white plaque with a slightly verrucous surface, approximately 3 cm in size, located on the left buccal mucosa (Fig. 1). Given the appearance of the lesion, the diagnostic hypothesis was leukoplakia. An incisional biopsy was performed, and smoking cessation instructions were given. Microscopic examination revealed high-grade epithelial dysplasia, corroborating the clinical diagnosis of leukoplakia (Fig. 2). Considering the lesion location and extent, the treatment of choice was complete excision using High-Power Diode Laser (TW Surgical Laser, MMOptics, São Carlos-SP, Brazil), delivering the beam with a 400 µm optical fiber, 808 nm wavelength (infrared) in continuous mode and 1.5 W power. The procedure was performed under local infiltrative anesthesia with 2% mepivacaine with vasoconstrictor (1.8 mL). For postoperative care, oral analgesics (1 g dipyrone every 12 hours in case of pain) and 0.12% chlorhexidine digluconate mouthwash, three times a day, for 14 days were prescribed. After seven days, the area appeared raw and painless (Fig. 3). After 30 days, no recurrence was observed, and complete healing of the area was noted. After 18 and 30 months (Fig. 4), clinical examination revealed no recurrence of the lesion. Despite instructions regarding smoking cessation, the patient still reports smoking, although less frequently. Therefore, continuous follow-up is essential.

**Fig. 1:**
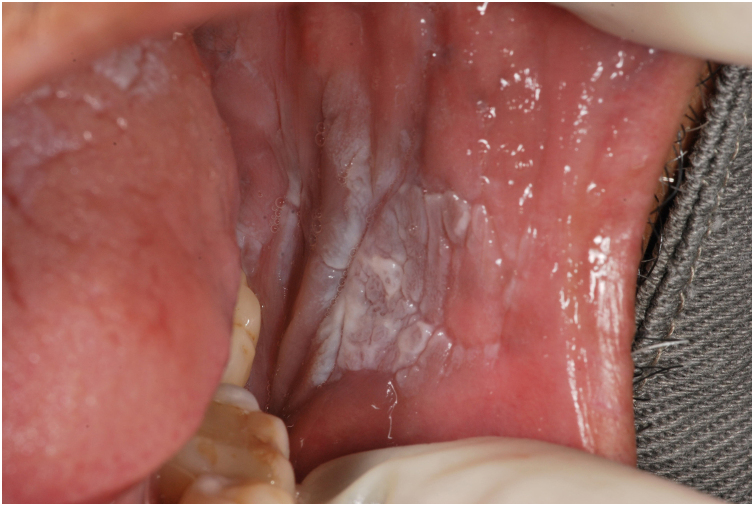
Initial image of the lesion in the buccal mucosa on the left side.

**Fig. 2:**
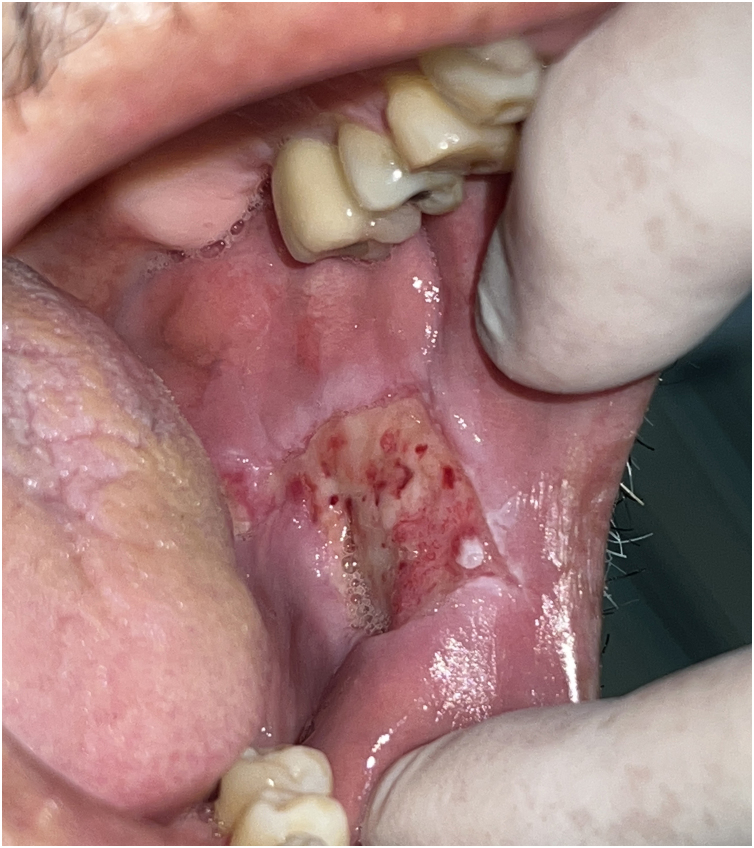
Representative image of healing seven days after surgery with a diode laser.

**Fig. 3:**
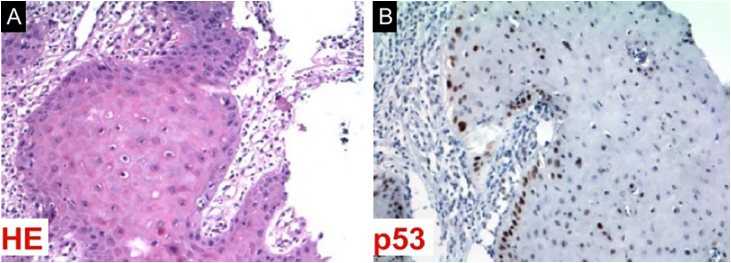
(A) Photomicrograph showing epithelium with high-grade dysplasia. (B) Immunohistochemical study revealing expression for the tumor suppressor gene TP53.

**Fig. 4:**
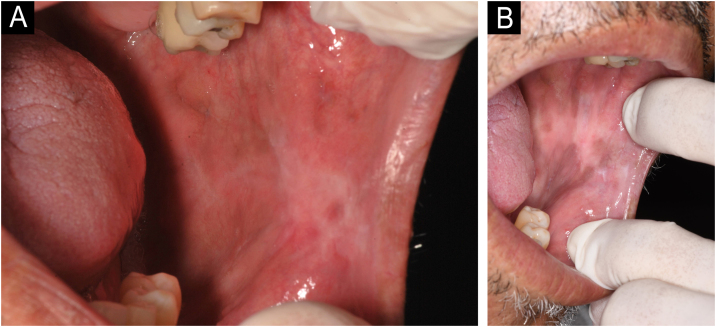
Appearance of the left buccal mucosa after 30 months. The pigmented areas are suggestive of melanosis due to smoking.

The literature is unanimous on the positive effects of lasers in oral surgeries, such as absence of mechanical trauma, adequate hemostatic capacity, visualization of the surgical field, antisepsis of the surgical wound, elimination of the need for sutures, and pain and edema reduction in the postoperative period.^4^, ^8^, ^9^, ^10^ The thermal impact of the surgical laser can cause the closure of blood and lymphatic vessels, minimizing the possibility of neoplastic cells being disseminated to other organs through circulation.^9^

In the present case report, the choice of the diode laser was mainly due to the extent and anatomical site of the lesion, aiming to provide the patient with the benefits this surgical technique offers. The scar evolution was carefully monitored, corroborating the postoperative advantages that the technique provides. In this case report, after 30 months, the clinical aspect did not reveal recurrences, suggesting the effectiveness of the surgical procedure and excellent prognosis.

Based on this case report, it can be suggested that the use of diode laser in selected cases of OL offers the possibility of removing extensive lesions in a single surgical procedure, with decontamination of the surgical field, residual photobiomodulation effect (modulation of inflammation, analgesia, and acceleration of healing), with excellent hemostasis and visualization of the surgical field, as well as reduced postoperative medication and greater patient comfort. However, the use of this technology is still limited due to the small number of qualified professionals and high cost.

## ORCID ID

Elen de Souza Tolentino: 0000-0002-4352-4694

Fábio Vieira de Miranda: 0000-0002-8188-1545

Tiago Carvalho dos Santos: 0000-0002-4276-4354

Lucas Hideki Suzaki Ikeshoji: 0009-0009-9786-9194

Letícia Sant’Anna Arioso: 0009-0002-2599-8615

Paulo Sérgio da Silva Santos: 0000-0002-0674-3759

Camila Lopes Cardoso: 0000-0001-9545-6809

## Financial support

None declared.

## Authors’ contributions

Elen de Souza Tolentino: Effective participation in the therapeutic conduct of the studied case; design and planning of the study; drafting and editing of the manuscript.

Fábio Vieira de Miranda: Effective participation in the therapeutic conduct of the studied case, interpretation of the data; approval of the final version of the manuscript.

Tiago Carvalho dos Santos: Effective participation in the critical review of the literature and the manuscript.

Lucas Hideki Suzaki Ikeshoji: Effective participation in the drafting and editing of the manuscript; critical review of the literature and the manuscript.

Letícia Sant’Anna Arioso: Effective participation in the critical review of the literature and the manuscript.

Paulo Sérgio da Silva Santos: Effective participation in the critical review of the manuscript, and approval of the final version of the manuscript.

Camila Lopes Cardoso: Effective participation in the drafting and editing of the manuscript and orientation of the article.

## Research data availability

Not applicable.

## Conflicts of interest

None declared.
